# High-Throughput Sequencing Indicates a Novel Marafivirus in Grapevine Showing Vein-Clearing Symptoms

**DOI:** 10.3390/plants10071487

**Published:** 2021-07-20

**Authors:** Xudong Fan, Zunping Zhang, Chen Li, Fang Ren, Guojun Hu, Baodong Zhang, Yafeng Dong

**Affiliations:** National Center for Eliminating Viruses from Deciduous Fruit Trees, Research Institute of Pomology, Chinese Academy of Agriculture Sciences, Xingcheng 125100, China; fanxudong@caas.cn (X.F.); zhangzunping@caas.cn (Z.Z.); caaslc@163.com (C.L.); renfang@caas.cn (F.R.); huguojun@caas.cn (G.H.); mayday0318143@163.com (B.Z.)

**Keywords:** grapevine, high-throughput sequencing, Marafivirus, vein-clearing, sequence analyses

## Abstract

A putative new marafivirus was identified in a ‘Jumeigui’ grapevine exhibitting obvious vein-clearing symptoms by high-throughput sequencing, which tentatively named grapevine-associated marafivirus (GaMV). The nearly complete genomic sequence of GaMV was amplified by reverse transcription PCR, and the terminal sequences were determined using the rapid amplification of cDNA ends method. The nearly complete genome of GaMV is 6346 bp long, excluding the poly(A) tail, and shows 51.2–62.3% nucleotide identity with other members of the genera *Marafivirus*, *Maculavirus* and *Tymovirus* in the family *Tymoviridae*. Additionally, it includes five functional domains homologous to those found in members of these genera. A phylogenetic analysis showed that GaMV clustered with other species-related marafiviruses. These data support GaMV being a representative member of a novel species in the genus *Marafivirus*. Furthermore, GaMV was graft-transmissible and 26 of 516 (5.04%) grapevine samples from five provinces in China tested positive by reverse transcription PCR. The coat protein of GaMV isolates shared 91.7–100% and 96.7–100% identities at the nt and aa levels, respectively. The coat protein-based phylogenetic trees revealed three well-defined clusters.

## 1. Introduction

The International Committee on the Taxonomy of Viruses divides the family *Tymoviridae* into three genera, *Maculavirus*, *Marafivirus* and *Tymovirus* [1]. These positive single-stranded RNA viruses contain genomes of approximately 6.0–7.5 kb. There are five viruses belonging to the family *Tymoviridae* that are reported to infect grapevine: Grapevine fleck virus (GFkV), grapevine red globe virus (GRGV), grapevine Syrah virus 1 (GSyV-1), grapevine asteroid mosaic-associated virus (GAMaV) and grapevine rupestris vein feathering virus (GRVFV). Among them, GFkV and GRGV belong to the genus *Maculavirus*, whereas GSyV-1, GAMaV and GRVFV belong to the genus *Marafivirus* [2]. GFkV, GRGV, GAMaV and GRVFV are associated with grapevine fleck complex, which is a major grapevine viral disease distributed worldwide [3]. This complex consists of several diseases, including grapevine fleck (FK), grapevine asteroid mosaic (AM), grapevine rupestris necrosis and grapevine rupestris vein feathering. The first recognized disease of this complex in California was AM [4]. It has been successfully transmitted by grafting and is characterized by translucent/chlorotic star-like spots on the foliage of several *Vitis vinifera* cultivars, as well as the clearing of primary and secondary veins of *Vitis rupestris* [5]. An isometric virus with morphological traits resembling those of GFkV was purified from AM-affected *V. rupestris* [5] and named GAMaV. GAMaV was further characterized after its genome was sequenced [6,7,8]. The FK disease has also been reported in California, and it induces symptoms on *V. rupestris* that are distinct from those of AM. It is characterized by the clearing of the third- and fourth-order veins [9,10]. In Italy, GFkV is associated with the FK-affected *V. rupestris* [11]. GRVFV is associated with vein feathering on *V. rupestris*, which is characterized by the transient mild chlorotic discoloration of the primary and secondary leaf veins [3,5]. In addition to the viruses causing these diseases, the grapevine vein-clearing virus is associated with grapevine vein-clearing symptoms, similar to those caused by AM [12].

During a field investigation in 2014, a disease causing obvious vein-clearing symptoms was observed on a ‘Jumeigui’ grapevine in Dalian City, Liaoning Province, China. The symptoms were similar to those of AM caused by the grapevine fleck complex. The possible agent of this disease was thought to be GAMaV or GCVC, but neither was detected by reverse transcription (RT)-PCR in this sample. In 2017, to identify possible viral infections in the diseased grapevine sample, small RNA sequencing (sRNA-seq) and RNA sequencing (RNA-seq) were employed.

## 2. Results

### 2.1. Analyses of the High-Throughput Sequencing (HTS) Data

**Figure 1:**
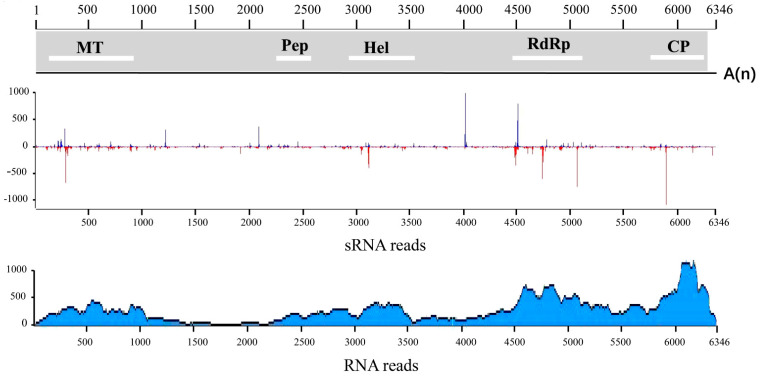
SRNA and RNA reads mapped to the genome of grapevine-associated marafivirus (GaMV). MT, methyltransferase; Pro, protease; Hel, helicase; RdRP, RNA−dependent RNA polymerase; CP, coat protein; A_(n)_, poly(A) tail.

Sequencing of sRNAs and RNAs from symptomatic leaves of the ‘Jumeigui’ grapevine resulted in 18, 599, 455 and 602, 231, 929 clean reads, respectively. The clean data were analyzed to identify viral sequences using VirusDetect software. The sRNA-seq data revealed 17 contigs of 55–286 nt that were homologous to polyproteins of GAMaV (AOX24075), representing 26.6% coverage (Figure S1), and the RNA-seq data revealed 14 contigs of 206–4866 nt that were homologous to polyproteins of oat blue dwarf virus (AAC57874), representing 94.6% coverage (Figure S1). These results indicated the presence of a candidate marafivirus in the ‘Jumeigui’ grapevine sample, which we tentatively named grapevine-associated marafivirus (GaMV). Mapping results showed that 50,789 sRNAs and 12,937 RNAs from sRNA-seq and RNA-seq clean data were derived from GaMV, respectively (Figure 1). Additionally, both sRNA-seq and RNA-seq identified some contigs in the sample that were homologous to sequences ofgrapevine geminivirus A, grapevine leafroll-associated virus-3 (GLRaV-3), grapevine rupestris stem pitting-associated virus, GFkV, grapevine Pinot gris virus, grapevine virus E, grapevine yellow speckle viroid 1, hop stunt viroid and citrus exocortis viroid.

### 2.2. Sequence and Phylogenetic Analyses of the GaMV Genome

Five overlapping fragments of GaMV isolate JMG were amplified by RT-PCR with specific primers and were assembled into a contiguous sequence by overlapping common regions (in general, approximately 100 bp) of the amplicons. Furthermore, we obtained the intact sequence of the 3′-untranslated regions (UTR) using 3′-rapid amplification of cDNA ends (RACE), and the partial sequences of 5′-end was obtained, after several attempts to produce a longer fragment, using 5′-RACE. Finally, we obtained the nearly complete genome of GaMV (GenBank accession NO. MZ422607), which was 6346 bp, excluding the polyA tail. The nearly complete GaMV sequence showed 51.2–62.3% identities with the genera *Marafivirus*, Maculavirus and *Tymoviruses* in the family *Tymoviridae* (Table 1). The following five domains were identified in the polyprotein using the CD search tool at the National Center for Biotechnology Information database (Figure 1): (i): A viral methyltransferase (Met) (at 113–958 nt), which is found throughout the Alphavirus superfamily and is involved in mRNA capping. It shared 57.2–70.2% nt and 50.0–70.2% aa identities with other members of *Tymoviridae*; (ii): tThe tymovirus endopeptidase proteolytic domain (Pep) (2264–2569 nt), which may mediate an additional cleavage between the protease and RNA helicase (Hel) domains of the polyprotein [13]. It shared 51.4–57.4% nt and 25.0–47.4% aa identities with other related viruses; (iii): The Hel domain (2,843–3,544 nt). In the N- terminal region of the GaMV Hel domain, a sequence, ^952^GFAGCGKS^959^, homologous to the NTP-binding site of RNA viruses was present. The Hel domain showed 57.3–70.7% nt and 50.0–70.2% aa identity levels with those of other related viruses; (iv): the RNA-dependent RNA polymerase domain (4490–5185 nt) shared 63.4–77.6% nt and 63.8–80.7% aa identities with those of other viruses; and (v): the tymovirus coat protein (CP) (5705–6223 nt) shared 39.8–68.2% nt and 22.0–63.4% aa identities with the CP of other tymoviruses. A marafibox-like sequence, CAGGGTGAATTGCTTCA, was found at 5501–5517 nt in the GaMV genome, and it may be the transcriptional promoter that drives the synthesis of a subgenomic RNA encoding only the CP translational reading frame [14].

**Figure 2:**
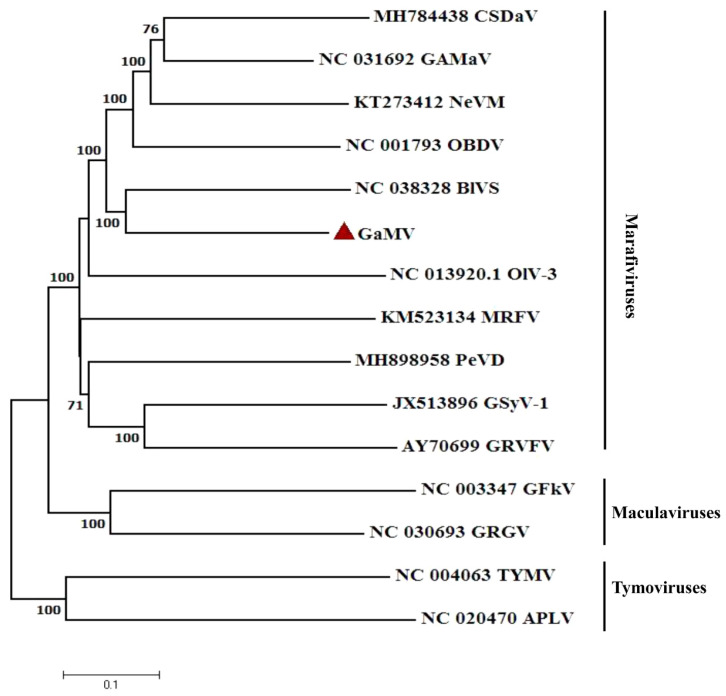
Neighbor-joining phylogenetic tree generated from the genomic sequences of grapevine-associated marafivirus (GaMV) and other members of the genera *Marafivirus*, *Maculavirus* and *Tymovirus* in the family *Tymoviridae*.

A phylogenetic tree was constructed to establish the relationships between GaMV and other members of the family *Tymoviridae* (Figure 2). The nucleotide sequences of the complete genomes from all the approved marafiviruses, and several members of the genera Maculavirus and *Tymovirus*, were used in the analyses. GaMV clustered together with marafiviruses with high bootstrap values, thereby confirming a close phylogenetic relationship between GaMV and members of the genus *Marafivirus* (Figure 2).

### 2.3. Graft-Transmission of GaMV

**Figure 3:**
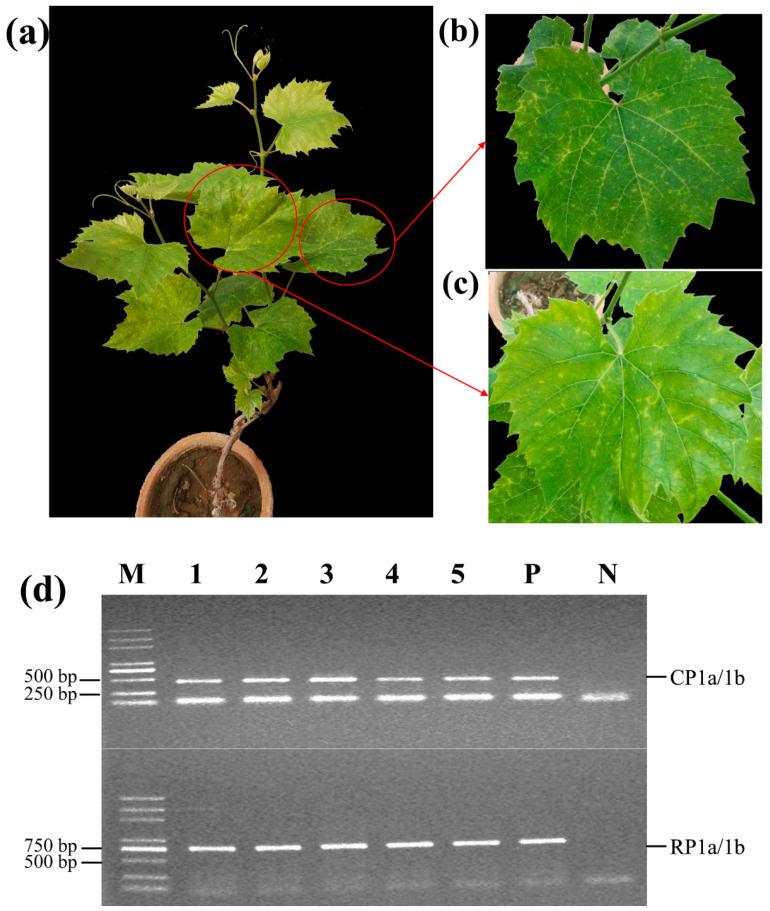
Testing the transmission of grapevine-associated marafivirus (GaMV) to grapevines. (**a**) a grafted ‘Beta’ grapevine; (**b**,**c**) partially enlarged images of (**a**); (**d**) Reverse transcription PCR (RT-PCR) detection of GaMV in five grafted grapevines using two sets of specific primers. Lane 1–5, grafted grapevine samples; P, positive control; N, negative control; M, DNA marker DL5000 (TaKaRa).

All the grafted ‘Beta’ grapevines showed obvious symptoms of vein-clearing and chlorotic mottling (Figure 3a–c). At 12 months after grafting, the five grafted ‘Beta’ grapevines tested positive for GaMV using the two specific PCR primer pairs (Figure 3d). In contrast, non-grafted plants tested negative for GaMV and did not show symptoms. These data suggested that GaMV was transmissible by grafting.

### 2.4. Detection of GaMV in the Field

RT-PCR assays were performed to detect GaMV in 516 samples, and 23 of 516 (4.46%) and 12 of 516 (2.33%) tested positive using the primers CP1a/1b and RP1a/1b, respectively (Table S1), suggesting that the former primer set had a better amplification capability than the latter primer set. In total, 26 of 516 grapevine samples (5.04%), collected from Beijing (1), Liaoning (21), Ningxia (2), Shandong (1) and Sichuan (1) provinces, tested positive for GaMV.

### 2.5. Sequence Identities of CP Genes and Phylogenetic Relationships between Different GaMV Isolates

**Figure 4:**
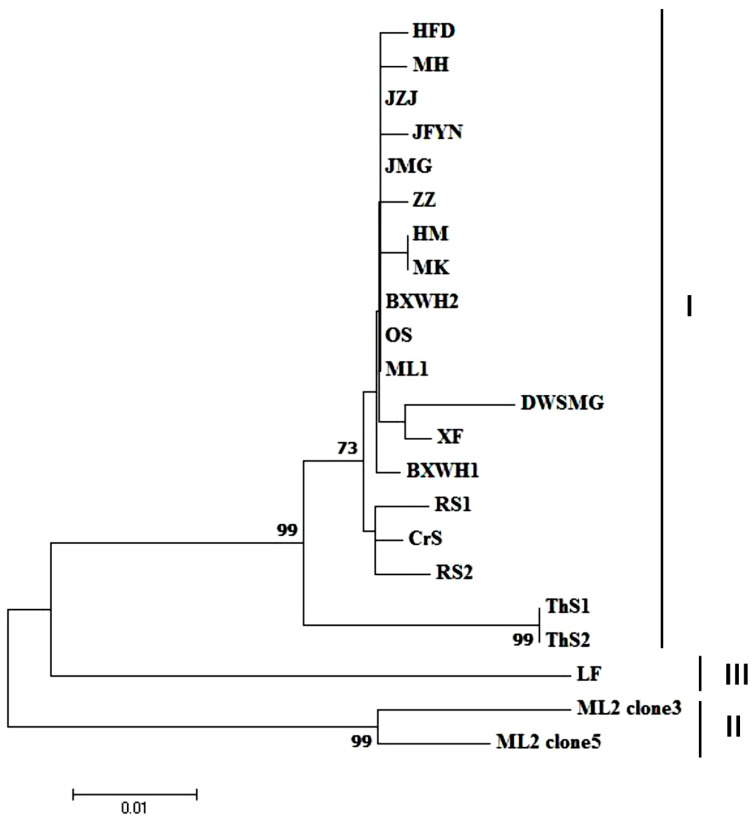
Neighbor-joining phylogenetic tree generated using CP gene sequences of grapevine-associated marafivirus (GaMV) isolates.

The *CP* sequences from 20 GaMV isolates were obtained to analyze the genetic diversity among GaMV isolates. The sequences of these isolates have been deposited in GenBank (Table 2). For all these isolates, except ML2, the three initially sequenced clones within a single grapevine showed >99.0% nt identity levels. Therefore, more clones of isolate ML2 were sequenced and two variant types, represented by ML2 clone 3 and ML2 clone 5, were identified. The CPs of GaMV isolates showed 91.7–100% and 96.7–100% identities at the nucleotide and amino acid levels, respectively (Table S2). The CP-based phylogenetic tree revealed the existence of three well-defined clusters. The major isolates belonged to group I, whereas isolates ML2 and LF belonged to groups II, and III, respectively (Figure 4).

## 3. Discussion

More than 80 species of grapevine viruses have been reported [15], to date, and new grapevine viruses continue to be identified, largely owing to HTS technology [16]. Most grapevine viruses are associated with the three major viral disease complexes in grapevine: Infectious degeneration, leafroll and rugose wood. The next most important disease is the grapevine fleck complex, which occurs worldwide [3]. Among the viruses related to the disease, GFkV, GRGV, GSyV-1 and GRVFV have been found in China, but their pathogenicity levels remain poorly understood. Here, we demonstrated an infection caused by a new marafivirus, GaMV, in a grapevine sample showing obvious vein-clearing symptoms. Our results provide new knowledge on the fleck complex caused by viruses in the genus *Marafivirus*.

The genomic sequences of GaMV in the diseased ‘Jumeigui’ grapevine were confirmed by sRNA-seq, RNA-seq and traditional Sanger sequencing of PCR products. The nearly complete genome of GaMV was also determined by multiple 5′-RACE amplifications, which produced the longest obtainable genomic fragments. The genomic sequence included domains homologous to all five common domains found in other marafiviruses, and it also contained a marafibox-like sequence similar to that conserved in viruses belonging to *Marafivirus* [14]. Furthermore, the complete genome-based phylogenetic tree revealed that GaMV clustered with other species-related marafiviruses. Additionally, this study demonstrated the graft transmission of GaMV. These characteristics strongly support GaMV being a novel member of the genus *Marafivirus*.

Vein clearing is a typical symptom of the grapevine fleck complex. Although the symptoms observed on the ‘Jumeigui’ samples were very similar to those of AM, we did not confirm a relationship between GaMV and the disease because the sample contained multiple viruses, including GFkV and grapevine Pinot gris virus. GFkV has been associated with the grapevine fleck complex, whereas GPGV has been putatively associated with a novel grapevine disease, known as grapevine leaf mottling and deformation [17]. Therefore, the pathogenicity of GaMV alone still needs to be determined. The field survey showed that GaMV was present in the field at a moderate rate (5.04%), and analyses of *CP* genes from different samples indicated that there were variants. Therefore, the potential harm of GaMV to grapevines is a cause for concern because of its graft transmissibility and occurrence status.

## 4. Materials and Methods

### 4.1. Plant Material for HTS

**Figure 5:**
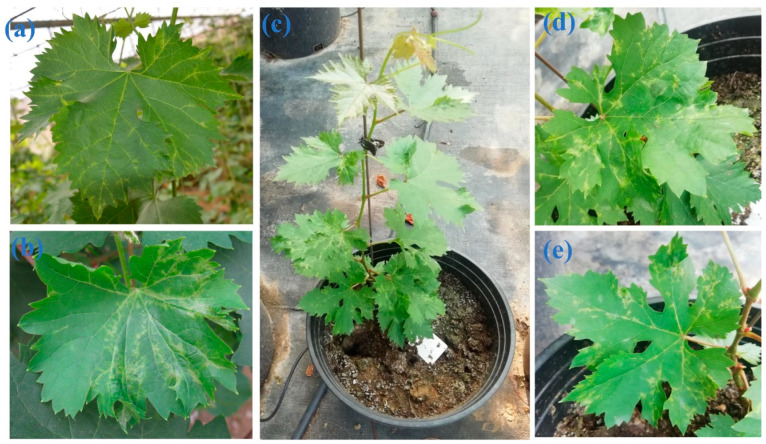
Symptoms on the leaves of a ‘Jumeigui’ grapevine infected with grapevine-associated marafivirus (GaMV). (**a**,**b**) vein-clearing symptoms on GaMV-infected grapevine leaves; (**c**) cut seedlings propagated from the GaMV-infected plant; (**d**,**e**) enlarged images of the diseased leaves of (**c**).

A ‘Jumeigui’ grapevine, showing vein-clearing symptoms (Figure 5a,b), was collected from a vineyard of the Dalian Academy of Agricultural Sciences (Dalian City, Liaoning Province, China) in 2014. Cutting propagated from the infected grapevine also showed vein-clearing symptoms (Figure 5c–e). In spring 2017, diseased leaves were collected and frozen rapidly in liquid nitrogen before being preserved in carbon dioxide ice-blocks and shipped to Biomarker Biology Technology (Beijing, China). Transport took 2–3 days.

### 4.2. HTS and Bioinformatics Analyses

Leaf samples were used to extract total RNAs and generate a cDNA library of sRNAs. sRNA-seq was carried out using an Illumina HiSeq™ 2000 system (SinoGenoMax, Beijing, China), as reported previously [18]. Clean data were obtained by removing sequences <18 nt or >30 nt, low-quality tags, poly-A-tags and N-tags from raw reads. Sequences of potential viruses were identified by analyzing the clean data using VirusDetect (http://virusdetect.feilab.net/cgi-bin/virusdetect/index.cgi/) [19]. For RNA-seq, the Epicentre Ribo-Zero rRNA Removal Kit (Epicentre, Madison, WI, USA) was used to remove ribosomal RNA from extracts of total RNA. The ribosomal RNA-depleted RNA sample was then used to construct a cDNA library using a TruSeq RNA Sample Prep Kit (Illumina, San Diego, CA, USA), which was sequenced on an Illumina HiSeq 4000 platform with a paired-end 150-bp format (Biomarker Biology Technology). Reads mapping to the grapevine genome (PN40024 assembly 12X) were removed by hierarchical indexing using hisat software [20]. Unmapped reads were used for de novo assembly and BLAST analyses embedded in VirusDetect.

### 4.3. Amplification and Analyses of the GaMV Genome

Five primer pairs were designed on the basis of contig sequences and were used to amplify GaMV genomic sequences (Table 3). PCR fragments were recovered, purified and then cloned into the Zero Background pTOPO-Blunt vector (Aidlab, Beijing, China). At least three positive clones of each PCR product were sequenced at Shanghai Sangon Biological Engineering & Technology (Shanghai, China). The 5′- and 3′-UTRs were amplified by the RACE strategy using a SMARTer^®^ RACE 5′/3′ Kit (TaKaRa) in accordance with the manufacturer’s instructions.

### 4.4. Sequence Analyses

The tool ORF Finder at the National Center for Biotechnology Information was used to search for potential open reading frames in the genomic RNA of GaMV. The CD search tool was used to search for conserved domains. The nucleotide and amino acid sequences were compared with those of other marafiviruses using ClustalW2 (www.ebi.ac.uk/Tools/msa/clustalw2/). For phylogenetic analyses, the representative members of the genera *Marafivirus*, Maculavirus and *Tymoviruses* in the family *Tymoviridae* were used. Neighbor-joining trees were constructed using Molecular Evolutionary Genetics Analysis (MEGA) 7.1 (www.megasoftware.net/).

### 4.5. Graft-Transmission Assays

Graft transmissibility was assessed in July 2019 by grafting GaMV-infected grapevine buds onto 2-year-old ‘Beta’ grapevine seedlings with five replicates. These seedlings had tested negative for GaMV and the following other major viruses reported in China: GLRaV-1, -2, -3, -4, -7 and -13, grapevine rupestris stem pitting-associated virus, GFkV, grapevine fanleaf virus, grapevine virus A(GVA), GVB, GVE, grapevine Pinot gris virus, grapevine berry inner necrosis virus, grapevine fabavirus, GRVFV, grapevine geminivirus A, GSyV-1 and GRGV. Inoculated seedlings were maintained in a greenhouse under natural conditions for symptom development. At 3, 6 and 12 months after inoculation, grafted grapevines were monitored continuously for symptom development. Total RNAs were extracted from the leaves of the grafted ‘Beta’ grapevines using the method described by MacKenzie and colleagues [21] with slight modifications [22]. Two primer pairs, RP1a/1b (5′-AAGACTCAGCACAAGGTCAACGAG-3′ and 5′-CACGGCTTCTACCGGGAGGAG-3′) and CP1a/1b (5′-GATTCTCGCATTGATGCTCAGC-3′ and 5′-TAGATGGTGTAGGAGTAGCGG -3′), were used to amplify the 780-bp fragment of the RdRP and the 458-bp fragment of the CP gene, respectively, which were used in the RT-PCR detection of GaMV in these samples.

### 4.6. Survey of GaMV in the Field Samples

To survey GaMV prevalence, 516 grapevines, representing 71 cultivars, were randomly collected from 21 provinces in China. Total RNA extraction and RT-PCR detection were carried out as described in Section 4.5. The PCR products that were assessed positive for the *CP* gene were cloned and sequenced, as described in Section 4.3.

## Figures and Tables

**Table 1:** Percentage identities for different polyprotein domains of representative viruses compared with grapevine-associated marafivirus (GaMV).

Virus	Genome		Met		Pep		Hel		RdRp		CP	
	Length	%nt	%nt	%aa	%nt	%aa	%nt	%aa	%nt	%aa	%nt	%aa
BlVS	6463	61.0	69.3	65.6	55.0	45.7	66.5	65.6	74.2	80.7	65.5	59.3
CSDaV	6805	58.3	67.5	65.6	51.8	38.3	66.5	65.6	72.4	79.3	64.5	61.5
GAMaV	6719	62.3	71.2	69.5	57.4	44.3	70.7	69.5	77.6	80.5	68.2	63.4
GSyV-1	6481	57.1	66.2	60.6	54.6	42.4	62.0	60.6	70.2	69.5	55.6	41.9
MRFV	6337	60.5	66.9	64.5	56.3	43.5	63.0	64.5	72.3	76.8	61.8	48.0
NeVM	6471	59.5	66.4	66.3	55.0	44.3	67.2	66.3	74.9	81.1	61.4	57.0
OBDV	6509	62.0	70.2	70.2	54.3	41.2	67.2	70.2	76.8	81.1	66.7	56.7
OlV-3	7148	55.3	62.9	62.4	54.4	47.4	58.3	62.4	70.6	72.7	56.1	52.0
PeVD	6573	56.6	66.9	63.5	54.6	44.4	63.0	63.5	72.8	80.2	54.3	39.6
GRVFV	6577	54.7	64.3	60.5	52.1	42.6	61.6	60.5	71.4	74.3	55.3	41.8
GFkV	7564	55.4	63.7	50.0	55.8	25.0	63.2	50.0	69.0	64.7	51.9	34.0
GRGV	6850	52.2	65.0	58.2	56.3	40.7	63.7	58.2	66.5	63.8	49.8	31.7
APLV	6337	51.2	60.5	55.9	51.4	37.2	59.1	55.9	63.5	63.8	40.5	28.0
TYMV	6318	52.5	57.2	54.3	53.1	36.5	57.3	54.3	63.4	64.4	39.8	22.0

Blackberry virus S, BlVS; citrus sudden death-associated virus, CSDaV; grapevine asteroid mosaic-associated virus, GAMaV; grapevine Syrah virus-1, GSyV-1; maize rayado fino virus, MRFV; nectarine marafivirus M, NeVM; oat blue dwarf virus, OBDV; olive latent virus 3, OlV-3; peach marafivirus D, PeVD; grapevine rupestris vein feathering virus, GRVFV; grapevine fleck virus, GFkV; grapevine red globe virus, GRGV; andean potato latent virus, APLV; and turnip yellow mosaic virus, TYMV.

**Table 2:** Details of grapevine-associated marafivirus (GaMV) isolates generated and analyzed in this study.

Isolate ID	Samples	location	GenBank Accession Number
BXWH1	Bixiangwuhe	Liaoning	MZ422608
HM	Heimi	Liaoning	MZ422609
HFD	Hafude	Liaoning	MZ422610
ZZ	Zizao	Liaoning	MZ422611
JZJ	Jinzaojing	Liaoning	MZ422612
DWSMG	Dengwasimeigui	Liaoning	MZ422613
ThS1	Thompson Seedless	Liaoning	MZ422614
MH	Muscat Hamburg	Liaoning	MZ422615
CrS	Crimson Seedless	Liaoning	MZ422616
RS1	Ruby Seedless	Liaoning	MZ422617
MK	Muscat Kyoho	Liaoning	MZ422618
Ths2	Thompson Seedless	Liaoning	MZ422619
JFYN	Jiafeiyinv	Liaoning	MZ422620
BXWH2	Bixiangwuhe	Liaoning	MZ422621
RS2	Ruby Seedless	Liaoning	MZ422622
OS	Otilia Seedless	Liaoning	MZ422623
LF	Lefu	Liaoning	MZ422624
XF	Xiagnfei	Liaoning	MZ422625
ML1	Merlot	Shandong	MZ422626
ML2	Merlot	Sichuan	MZ422627-28

**Table 3:** Primers designed for PCR amplification of the grapevine-associated marafivirus (GaMV) genome.

Primer Name	Primer Sequence (5′→3′)	Position
GaMV-1F	ACCATCCACCGGGACACCATC	38–58
GaMV-1R	ATGTAGGGGATGGAAGAGCTC	1564–1544
GaMV-2F	ACCCGCCTTCCTCTGGGCTTG	1450–1470
GaMV-2R	GTGGCGCGGAAGTTGAAGAAG	2445–2425
GaMV-3F	GAGGATCTCTGGTCCGCTCTC	2318–2338
GaMV-3R	GGCGGTCGAGAAGAATGTAGC	3470–3450
GaMV-4F	ATCCTGACCAACTCGCAGAAC	3350–3370
GaMV-4R	GGCTCGAAATCAAGGACGGAG	5007–4987
GaMV-5F	CACTCACCTGCATGCGGCTCA	4863–4883
GaMV-5R	GTAGAAGGAGGTTTCGGTGCC	6001–5981
GaMV 5′-outer	TCTGAAGAAAGTCATGGCCGG	180–160
GaMV 5′-inner	AACGATGAGGCGTTGATGCCG	159–139
GaMV 3′-outer	TCCGCCTTCATCACCGACGAC	5804–5824
GaMV 3′-inner	TCTGAAGAAAGTCATGGCCGG	5842–5862

## Data Availability

All data have been presented in the manuscript and supplementary materials, so the study did not report other data.

## Supplementary Materials

The following are available online at https://www.mdpi.com/article/10.3390/plants10071487/s1, Figure S1: Contigs from sRNA-seq; and (**a**) RNA-seq data (**b**) mapped to the reference genomes of two marafiviruses, Table S1: The grapevine samples tested positive for grapevine-associated marafivirus (GaMV) using the primers CP1a/1b and RP1a/1b, Table S2: Sequence identities of CP genes among different grapevine-associated marafivirus (GaMV) isolates.
