# Supplementary material for: High-Throughput Sequencing Indicates a Novel Marafivirus in Grapevine Showing Vein-Clearing Symptoms
Source: Plants (Basel). 2021 Jul 20;10(7):1487. doi: 10.3390/plants10071487 (PMC8309299; doi:10.3390/plants10071487)
Supplement: Supplementary file 1 [file plants-10-01487-s001.zip › plants-1292996-supplementary.pdf]

plants MDPI

Supplementary materials

Xudong Fan, Zunping Zhang, Chen Li, Fang Ren, Guojun Hu, Baodong Zhang and Yafeng Dong *

National Center for Eliminating Viruses from Deciduous Fruit Trees, Research Institute of Pomology, Chinese Academy of Agriculture Sciences, Liaoning, Xingcheng 125100, China; fanxudong@caas.cn (X.F.); zhangzunping@caas.cn (Z.Z.); caaslc@163.com (C.L.); renfang@caas.cn (F.R.); huguojun@caas.cn (G.H.); mayday0318143@163.com (B.Z.); dongyafeng@caas.cn (Y.D.)

* Correspondence: dongyafeng@caas.cn; Tel.: +86-0429-0359-8278

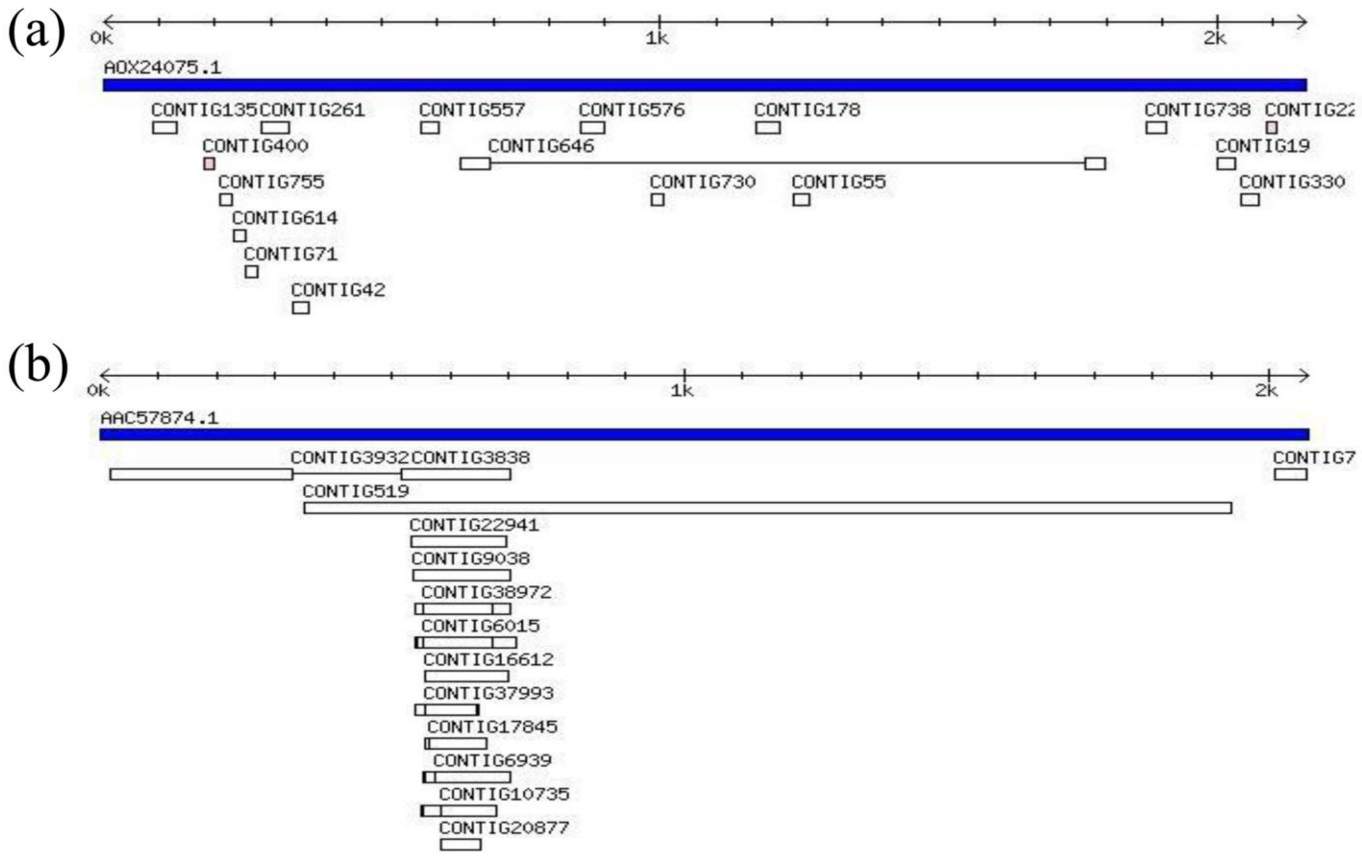

Figure S1. Contigs from sRNA-seq; and (**a**) RNA-seq data (**b**) mapped to the reference genomes of two marafiviruses.

Table S1. The grapevine samples tested positive for grapevine-associated marafivirus (GaMV) using the primers CP1a/1b and RP1a/1b.

Samples	location	CP1a/1b	RP1a/1b
Merlot	Beijing	+	-
Bixiangwuhe	Liaoning	+	-
Heimi	Liaoning	+	+
Hafude	Liaoning	+	-
Zizao	Liaoning	+	-
Jinzaojing	Liaoning	+	-
Dengwasimeigui	Liaoning	+	-
Thompson Seedless	Liaoning	+	-
Nanhalisi	Liaoning	-	+
Muscat Hamburg	Liaoning	+	+
Crimson Seedless	Liaoning	+	-
Ruby Seedless	Liaoning	+	+
Muscat Kyoho	Liaoning	+	+
Thompson Seedless	Liaoning	+	-
Jiafeiyinv	Liaoning	+	+
Bixiangwuhe	Liaoning	+	+
Ruby Seedless	Liaoning	+	+
Otilia Seedless	Liaoning	+	-
Rose couitat	Liaoning	+	+
Lefu	Liaoning	+	-
Xiagnfei	Liaoning	+	+
Fenghou	Liaoning	+	-
Cabernet Sauvignon	Ningxia	-	+
Cabernet Gernischt	Ningxia	-	+
Merlot	Shandong	+	-
Merlot	Sichuan	+	-
**The number of positive samples**		23	12
**Total number of positive samples**		26	

Table S2. Sequence identities of CP genes among different grapevine-associated marafivirus (GaMV) isolates.

	**JMG**	**BXWH1**	**HM**	**HFD**	**ZZ**	**JZJ**	**DWSMG**	**ThS1**	**MH**	**CrS**	**RS1**	**MK**	**ThS2**	**JFYN**	**BXWH2**	**RS2**	**OS**	**LF**	**XF**	**ML1**	**ML2 clone3**	**ML2 clone5**
JMG	100	99.3	100	100	99.3	100	100	98.0	99.3	100	100	100	98.0	100	100	98.7	100.0	99.3	100	100	98.7	98.7
BXWH1	99.8	100	99.3	99.3	98.7	99.3	99.3	97.4	98.7	99.3	99.3	99.3	97.4	99.3	99.3	98.0	99.3	99.3	99.3	99.3	98.7	98.7
HM	99.8	99.6	100	100	99.3	100	100	98.0	99.3	100	100	100	98.0	100	100	98.7	100	99.3	100	100	98.7	98.7
HFD	99.8	99.6	99.6	100	99.3	100	100	98.0	99.3	100	100	100	98.0	100	100	98.7	100	99.3	100	100	98.7	98.7
ZZ	99.8	99.6	99.6	99.6	100	99.3	99.3	97.4	98.7	99.3	99.3	99.3	97.4	99.3	99.3	98.0	99.3	98.7	99.3	99.3	98.0	98.0
JZJ	100	99.8	99.8	99.8	99.8	100	100	98.0	99.3	100	100	100	98.0	100	100	98.7	100	99.23	100	100	98.7	98.7
DWSMG	99.6	99.3	99.3	99.3	99.3	99.6	100	98.0	99.3	100	100	100	98.0	100	100	98.7	100	99.3	100	100	98.7	98.7
ThS1	97.6	97.4	97.4	97.4	97.4	97.6	97.2	100	97.4	98.0	98.0	98.0	100	98.0	98.0	96.7	98.0	97.4	98.0	98.0	96.7	96.7
MH	99.8	99.6	99.6	99.6	99.6	99.8	99.3	97.4	100	99.3	99.3	99.3	97.4	99.3	99.3	98.0	99.3	98.7	99.3	99.3	98.0	98.0
CrS	99.6	99.3	99.3	99.3	99.3	99.6	99.1	97.2	99.3	100	100	100	98.0	100	100	98.7	100	99.3	100	100	98.7	98.7
RS1	99.3	99.1	99.1	99.1	99.1	99.3	98.9	96.9	99.1	99.3	100	100	98.0	100	100	98.7	100	99.3	100	100	98.7	98.7
MK	99.8	99.6	100	99.6	99.6	99.8	99.3	97.4	99.6	99.3	99.1	100	98.0	100	100	98.7	100	99.3	100	100	98.7	98.7
ThS2	97.6	97.4	97.4	97.4	97.4	97.6	97.2	100	97.4	97.2	96.9	97.4	100	98.0	98.0	96.7	98.0	97.4	98.0	98.0	96.7	96.7
JFYN	99.8	99.6	99.6	99.6	99.6	99.8	99.3	97.4	99.6	99.3	99.1	99.6	97.4	100	100	98.7	100	99.3	100	100	98.7	98.7
BXWH2	100	99.8	99.8	99.8	99.8	100	99.6	97.6	99.8	99.6	99.3	99.8	97.6	99.8	100	98.7	100	99.3	100	100	98.7	98.7
RS2	99.3	99.1	99.1	99.1	99.1	99.3	98.9	96.9	99.1	99.3	99.1	99.1	96.9	99.1	99.3	100	98.7	98.0	98.7	98.7	97.4	97.4
OS	100	99.8	99.8	99.8	99.8	100	99.6	97.6	99.8	99.6	99.3	99.8	97.6	99.8	100	99.3	100	99.3	100	100	98.7	98.7
LF	93.4	93.4	93.2	93.2	93.2	93.4	93.2	92.4	93.2	93.4	93.7	93.2	92.4	93.2	93.4	93.2	93.4	100	99.3	99.3	99.3	99.3
XF	99.8	99.6	99.6	99.6	99.6	99.8	99.3	97.4	99.6	99.3	99.1	99.6	97.4	99.6	99.8	99.1	99.8	93.7	100	100	98.7	98.7
ML1	100	99.8	99.8	99.8	99.8	100	99.6	97.6	99.8	99.6	99.3	99.8	97.6	99.8	100	99.3	100	93.4	99.8	100	98.7	98.7
ML2 clone3	92.8	92.8	92.6	92.6	92.6	92.8	92.6	91.9	92.6	92.8	92.6	92.6	91.9	92.6	92.8	92.6	92.8	91.7	92.8	92.8	100	100
ML2 clone5	93.4	93.4	93.2	93.2	93.2	93.4	93.2	92.1	93.2	93.4	93.2	93.2	92.1	93.2	93.4	93.2	93.4	91.9	93.2	93.4	97.6	100

Note: nt identity between different GaMV isolates in the lower left; aa identity in the upper right.
